# Numerical Research on Supporting Component Defect Detection of Aramid Composite Honeycomb Structure by THz-TDS System

**DOI:** 10.3390/s25226910

**Published:** 2025-11-12

**Authors:** Pingan Liu, Xiangjun Li, Yongli Liu, Liguo Zhu

**Affiliations:** 1 Institute of Fluid Physics, China Academy of Engineering Physics, Mianyang 621900, China; liupingan23@gscaep.ac.cn (P.L.); liuyongli20@gscaep.ac.cn (Y.L.); 2 Centre for THz Research, China Jiliang University, Hangzhou 310018, China; xiangjun_li@cjlu.edu.cn

**Keywords:** aramid paper, honeycomb cell, THz-TDS, reflection, FDTD

## Abstract

The aramid honeycomb composite material plays an important role in industry. Defects of this material seriously influence its performance. However, conventional detecting tools such as X-ray or computer tomography (CT) imaging, ultrasonic testing, and visual inspection are not able to meet the requirements of fast, safe, and high resolution at the same time. In this study, we numerically use rapid terahertz time−domain spectroscopy (THz-TDS) to identify defects in the aramid paper composite structure effectively. Simulation results demonstrate that THz-TDS technology enables the non-destructive reflection imaging of layered defects in glass fiber covering and glue layers as supporting components within the composite structure, with a spatial resolution of 0.5 mm and a depth range exceeding 10 mm. During the study, the finite difference time domain (FDTD) simulation with a real pulse waveform is achieved, and the defect position can be recognized by the anomaly in the reflection profile when compared with the waveform reflected by non-defect samples. At the same time, it is found that the defect identification ability is obviously affected by the incident position. The numerical research illustrates that the detectable defect is as thick as 0.1 mm and has a diameter of 1 mm. The results will offer valuable guides to the real application of THz-TDS systems in the detection of a similar structure.

## 1. Introduction

A popular sandwich structure with two aramid honeycomb layers and three glass fiber covers has been widely applied in extreme environments, such as aviation, aerospace, and vehicle engineering, due to its high strength, flame retardant, low density, and high stiffness to weight ratio, meanwhile with the properties of low expansion coefficient, electro-magnetic wave and mechanical energy absorbing [1,2,3]. However, structural defects, such as voids, delamination, and inhomogeneous resin, will generate a huge impact on their mechanical and electromagnetic performance. The popular defect-detecting methods include X-ray/CT imaging, ultrasonic testing, and visual inspection. X-ray inspection has strong penetrating power and high resolution, but faces radiation hazards. Ultrasonic testing similarly possesses high penetration, resolution, and high safety. Nevertheless, it needs a coupling medium and has a blind zone. On the other hand, visual inspection holds high speed and accuracy. However, it lacks penetrating ability [4].

Recently, terahertz spectroscopy, an emerging non-destructive testing technology, has demonstrated a strong ability to detect and image internal defects in composite materials. THz waves are characterized by small scattering, high imaging resolution, strong penetration ability in non-polar materials, low electronic energy, and higher safety [5]. Especially, THz-TDS, a coherent detection technology that uses THz pulse amplitude and phase information, can detect tiny refractive index changes at the defect location, resulting in good imaging contrast for defects [6].

Numerous studies have been conducted to detect defects in composite materials using THz technology. Xing et al., adopting the reflection mode THz-TDS, detected several polymethacrylimide foam defects like debonding, crack, and void [6,7]. Yang et al. indicated the THz imaging accuracy for defects in composite materials with a deep learning algorithm [8]. Liu et al. showed research on the application of THz in detecting internal defects in foam sandwich panels [9]. Zhang et al. designed and built a portable continuous wave THz imaging system for detecting the space shuttle foam insulation [10]. Roth et al., using THz computed tomography, analyzed the voids and impact damage of the space shuttle’s external fuel tank [11].

However, a similar application of THz for detecting internal defects in thick and more complex structures, such as aramid honeycomb sandwich panels, is still lacking exploration. Particularly, the honeycomb layer structure with two aramid-paper layers is more complicated compared to a single aramid-paper layer [12,13,14] or simple foam sandwich structures, because its hexagonal period structure and panel supporting components affect THz waves with multiple refractions and reflections in different media layers. Therefore, it is important to first verify the feasibility of THz-TDS in non-destructive detection of defects in fiberglass panels and resins within the two aramid-paper layers’ honeycomb structure. It will bring a solid foundation for future experiment tests. Based on the FDTD method and a real waveform of the THz incident, the influences of the defects’ positions and shapes in panels are calculated. The numerical study demonstrates that the detectable defect is as thick as 0.1 mm and has a diameter of 1 mm. The results lay the foundation for practical applications of THz non-destructive testing in sandwich aramid honeycomb structures in the future.

## 2. Structures and Methods

**Figure 1:**
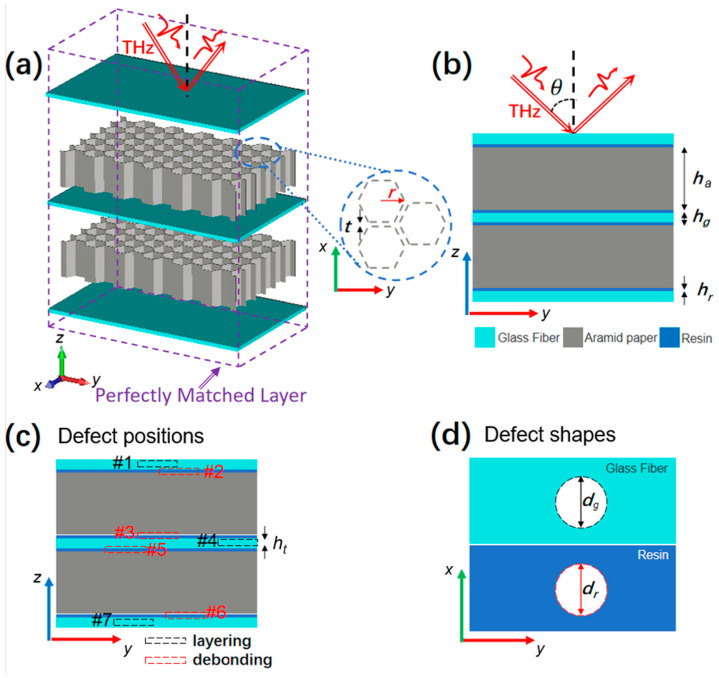
The sandwich structure scheme with two aramid honeycomb layers and three GFRP panels with defects. (**a**) 3D view; (**b**) side view; (**c**) defect positions; (**d**) defect shapes.

In this paper, a popular aramid honeycomb sandwich panel is mainly composed of two layers of aramid honeycomb cores (gray color) and three panels made of glass-fiber-reinforced plastic (GFRP), depicted in Figure 1a. The two core layers with void honeycomb structures in the aramid paper are reinforced by three panels with four layers of resin (blue color), shown in Figure 1b. It has been proven that the transmission of the THz pulse cannot indicate the defect positions; however, the varied reflected pulses at different times correspond to the shapes and positions of different defects [6]. Within a THz-TDS testing system operating in reflection mode, a picosecond THz pulse, represented as a Gaussian beam (red arrow), is incident on the top panel at an angle *θ*. The radius of the void hexagon pillar is *r*, and the aramid paper wall thickness of the honeycomb is *t*. Figure 1b illustrates that the *h_a_*, *h_g_*, and *h_r_* are the heights of the honeycomb core layer, the GFRP panel, and the resin layer. The values for the parameters based on the applied material size in a real scenario are *r* = 2.7 mm, *t* = 250 μm, *h_a_* = 10 mm, *h_g_* = 1 mm, and *h_r_* = 100 μm. The defect types we investigated here are the layering in the panels and the debonding in the resin. Figure 1c demonstrates that the position numbers #1~#7 denote different defects at the specific panel and resin layers. To simplify the study, the defect shapes are assumed to be thin void disks shown in Figure 1d. In the following research, the diameters of the defect disks filled with air in the panel and the resin will be changed by several millimeters, and the thickness of the void disk is set as 100 μm. Here, the deformation and damage defects of aramid honeycomb cores are not considered at present because the situation is more complicated and will be discussed in the future.

To numerically investigate the defect-detection capability of THz-TDS technology, the FDTD method is utilized to simulate the reflection of THz pulses from the sandwich structure with void disks in panels and resins at different positions. A box-like space with dimensions 12 mm × 12 mm × 20 mm is set up in the simulation with perfectly matched layers in six directions, as shown in Figure 1a. A THz pulse generated by the THz-TDS system [15,16] is incident on the top surface of the sandwich structure at an angle *θ*. The THz beam with an electrical field along the x-axis is incident on the structure, and the dielectric models of aramid paper, GFRP, and resin are taken from the references [17,18,19]. Under the normal incident, the time difference (∆t) between the surface reflection wave and the defect in the same material can be calculated or measured by considering the time delay due to the defect position *l* and the T-ray propagation path in reflection mode [20]:

(1) $$∆t=\frac{2l}{v}$$

where *v* refers to the T-ray speed inside the sample, which is determined by the T-ray speed in the air and the refractive index of the material.

**Figure 2:**
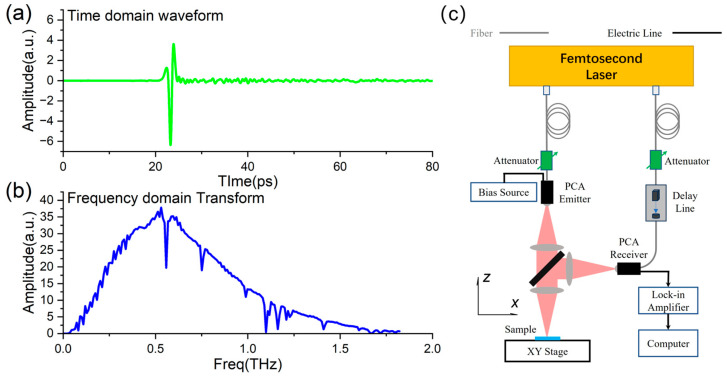
The THz-TDS pulse waveform and system for aramid honeycomb sandwich defect detection. (**a**) The real THz pulse waveform; (**b**) Frequency domain signal of the THz pulse for the simulation; (**c**) THz-TDS system with fiber coupling technology at reflection mode.

However, Equation (1) does not apply to the multilayered structure. The full-wave simulation, such as FDTD, is necessary. Here, the simulation time is 300 ps, which is long enough to cover all the reflection peaks of defects from the sandwich structure. To reproduce the measurement results of a real measurement system, a real waveform is applied in the research, as shown in Figure 2a, and its frequency domain transform is illustrated in Figure 2b. To improve the reflection detection accuracy and measurement flexibility, a system adopts a fully optical fiber design and works in a vertically reflecting configuration, which is theoretically demonstrated in Figure 2c. It is suitable for high-precision time−domain spectral analysis when the THz pulse effectively penetrates the structure with a honeycomb hole array. The principle of THz-TDS is to split the femtosecond laser beam into a strong pump beam and a weak probe beam by a beam splitter. The pump beam passes through a fiber and then reaches a THz radiation source, a photoconductive antenna (PCA), to excite THz pulses. After a collimating lens, the THz pulse penetrates the high-resistance silicon plate and is focused on the sample. And the reflected THz pulse returns to the focusing lens, then is reflected by the silicon plate. The second reflected pulse is focused on the receiving PCA, where it meets the probe beam, which passes through a time delay line. By adjusting the time delay line, the overall THz pulse waveform of the sample will be determined in the time domain. The delayed line rapid adjustment technology can achieve measurement speeds at the millisecond level, which enables fast scanning operation and imaging or tomography of the defect in two or three dimensions.

The lens in the system, designed for collimation and focusing, has a diameter of 25 mm and a focal length of 50 mm. The lateral resolution of the imaging system is approximately 1.5 mm. In the simulation, the scanning step is set to 0.5 mm. In the x-direction, the sample is moved forward and backward using a motorized stage, and in the y-direction, the sample is raster-scanned. The pixels 15*15 will be numerically generated at the x-y plane in the study. The reflection signal in each pixel has a 300 ps duration waveform. The simulated results can build a three-dimensional matrix that reveals the details of the defects’ positions and shapes.

## 3. Results and Discussions

**Figure 3:**
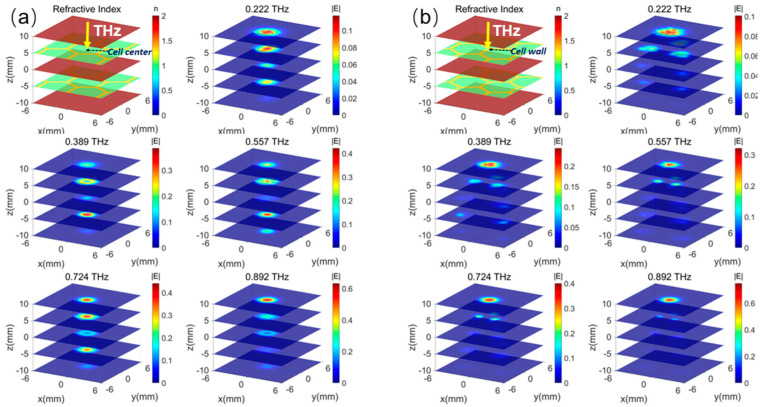
The electrical field distributions of the non-defect honeycomb sandwich structure at several intersections and frequencies. (**a**) THz incident beam at the cell center; (**b**) THz incident beam at the honeycomb cell wall.

To reveal the imaging mechanism of the detection system based on the THz-TDs technology, the field distributions of different layers without defects at several frequencies are first demonstrated in Figure 3. Although the fields cannot be measured in the experiments, this is valuable for analyzing the defect-detecting mechanism by showing how the THz pulse propagates in the structure. The incident position of the THz beam on the top GFRP panel is significant for its detecting ability due to the complex aramid honeycomb structure. In Figure 3a, when a THz incident beam is set at the honeycomb cell center, the THz Gaussian beam with a 1.5 mm diameter easily passes through the multilayer sandwich structure. Here, the indices of aramid paper, GFRP, and air are set as about 1.2, 1.5, and 1 around 0.5 THz, respectively, based on their real dielectric models. The simulated frequency points are 0.222 THz, 0.389 THz, 0.557 THz, 0.724 THz, and 0.892 THz, spanning a distance range from −10 mm to 10 mm in 5 mm increments. The maximum density increases gradually as the frequency grows. Except for 0.892 THz, where the energy mainly exists on the top GFRP panel, the fields are effectively penetrating the two honeycomb core layers at 0.222 THz, 0.389 THz, 0.557 THz, and 0.724 THz. On the other hand, in Figure 3b, the THz beam is incident on the cell wall where the field is fully scattered by the aramid paper, and most of the energy is trapped on the top floor at the same time. Similarly, the maximum density of the THz field *|E|* on the top panel is gradually increasing as the frequency increases.

Furthermore, it is assumed that there is a thin void defect disk in the center position of the center panel, which is the most difficult case to detect in the real scenario when the structure is subject to repeated twists and turns. We calculate the field distributions of different layers at several frequencies, shown in Figure 3. Obviously, when the THz beam is incident on the cell center in Figure 3a, the fields are altered at all layers and frequencies, while varying trends are similar to those of the structure without the defect. However, the field densities in the defect area are enlarged, especially at 0.557 THz. At the same time, Figure 3b illustrates the situation where the THz beam is incident on the cell wall. In this case, the field distributions are almost not varied, because the THz wave is fully scattered and trapped on the top GFRP panel. And the propagation of the THz wave is blocked by the cell wall. There is nearly no field energy entering the defect position, and the defect will not disrupt the field dispersion. Therefore, it is not possible to find the defect in this situation.

**Figure 4:**
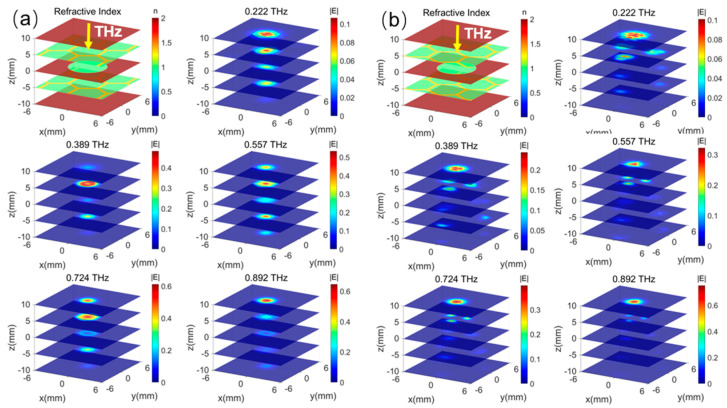
The electrical field distributions of the defect in the middle glass fiber (#4) of the honeycomb structure (diameter 6 mm) at different intersections and frequencies at the x-y plane. (**a**) THz incident beam at the cell center; (**b**) THz incident beam at the cell wall.

**Figure 5:**
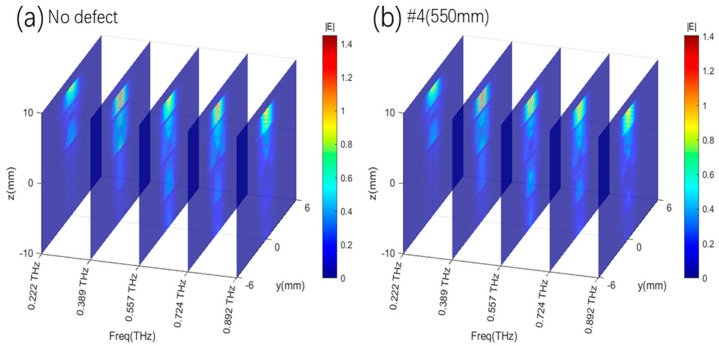
The electrical field distributions of the defect honeycomb structure (diameter 6mm) at different intersections and frequencies at the x-y plane. (**a**) THz incident beam at the cell center; (**b**) THz incident beam at the cell wall.

In the initial study, Figure 3 and Figure 4 only represent the field distributions on the x-y plane. Here, we also simulate the field at the above frequencies spreading on the y-z plane with x = 0 when the THz beam is input at the center of the honeycomb cell with no defect and a defect #4(550 μm), which, as the most difficult case for detection, is our main interest in Figure 5. The outcomes show the varied penetrating abilities of the THz wave at different frequencies. The THz wave at 0.557 THz has stronger penetrability than the other four frequencies along all layers, while most of the field densities are located at the honeycomb core layers along the z-axis. Furthermore, the fields are limited within the honeycomb cell along the y-axis for the waveguide effect formed by the aramid paper walls.

**Figure 6:**
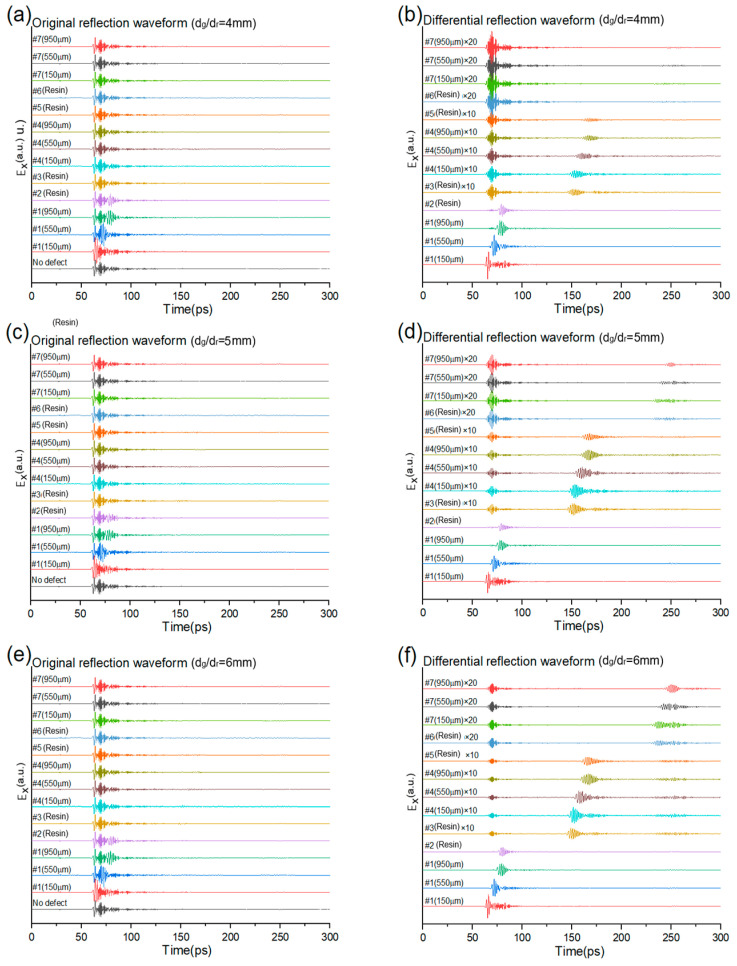
The cell center reflection waveforms of the honeycomb structure with the defects at the different positions. (**a**,**c**,**e**) Original reflection waveforms of non-defect or the 4 mm~6 mm diameter and 100 um thick middle layer defect. (**b**,**d**,**f**) Differential reflection waveforms of the defect by subtracting the non-defect structure.

In the next step, the real reflection waveforms of the input THz pulses are simulated, which carry information about the defect shapes and positions, as the corresponding refractive index change specifically alters the feature reflection waveform. Figure 6 demonstrates the reflection waveform of the different-sized defects at different positions of the structure with the THz incident at the cell center of the top GFRP panel. Figure 6a,c,e are the original simulated reflection waveforms of 100 μm thick void disk defects with diameters of 4 mm, 5 mm, and 6 mm. There are 14 time−domain curves representing various situations, including no defect and defects located at the three GFRP panels (#1, #4, and #7) at different depths (150 μm, 550 μm, and 950 μm from the top surface of each panel), as well as at four-layer resins (#2, #3, #5, and #6). The results show that there are obvious distortions when defects are located at the top panel and resin, but the feature reflections of the middle and button defects are very weak in this area. To improve the signal significance and intensity, the differential signals are computed by subtracting the non-defect signal from the defect signals, then enlarged by 10 (for defects #3~#5) or 20 (for defects #6, #7) times their amplitudes. The corresponding differential signals are plotted in Figure 6b,d,f for defects at the different positions with diameters of 4 mm, 5 mm, and 6 mm. With the above operation, the feature reflection pulses generated by the specific defects at different depth positions are arranged in order of time intervals. The results show that the depth of the defect can be uniquely determined by the time position of the specific reflection pulse. The amplitudes of the reflections decrease as the defect depth increases for the fixed-size defect, but they increase as the defect diameter grows from 4 mm to 6 mm. The amplitude envelopes are obviously varied between the void disk in the GFRP panel and the resin layers, while those in the panels are larger than in the resin layer when their positions are close, like #3, #4, and #5. These phenomena indicate that the amplitude and shape of the specific reflection pulse can be used to infer the size and profile of the defect.

To quantify the pulse-echo signals for different defect sizes, we also listed the peak times and amplitudes of the defects in Table 1, assuming the defect of #1 (150 μm) as a reference for each diameter. Here, it is clear that the echo peak time at the same position for 4 mm, 5 mm, and 6 mm is very close. The amplitudes decrease rapidly as the distance from the defect increases. The amplitudes of the defect in the middle and bottom layers are relatively small and hard to detect under a limited signal-to-noise ratio in the real scenario.

**Figure 7:**
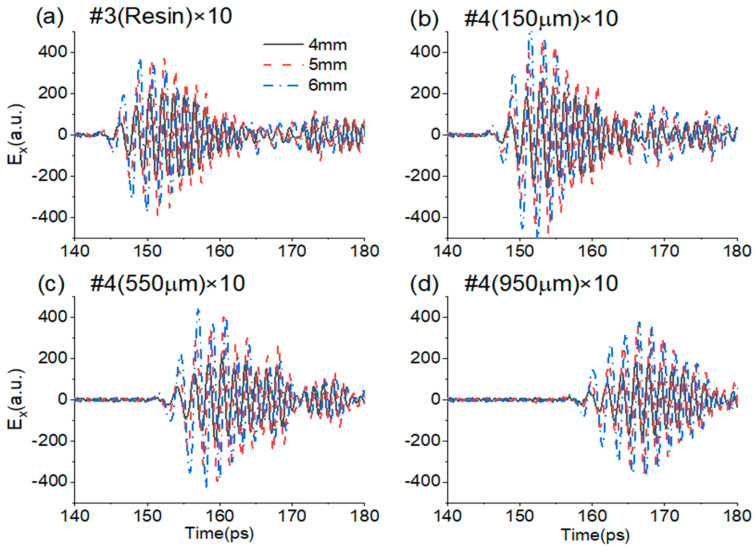
Comparisons of the differential reflection waveforms of void disk defects at the middle layer with different diameters of 4 mm, 5 mm, and 6 mm. (**a**) #3 (Resin) × 10; (**b**) #4 (150 μm) × 10; (**c**) #4 (550 μm) × 10; (**d**) #4 (950 μm) × 10.

It is clear that the defects in the near-surface layer are easier to detect, while those in the middle layer are more difficult to sense. To further solidify the ability of THz-TDS reflection measurement for determining the positions and shapes of middle-layer defects, comparisons of the reflection waveforms of void disks with different diameters of 4 mm, 5 mm, and 6 mm are made in Figure 7. The amplitudes of 5 mm and 6 mm disks are obviously larger than that of the 4 mm disk in the whole envelope. The start position of the glue defect #3 is about 3 ps ahead of the first GFRP defect #4 (150 μm), which is about 5 ps ahead of the second GFRP defect #4 (550 μm).

**Figure 8:**
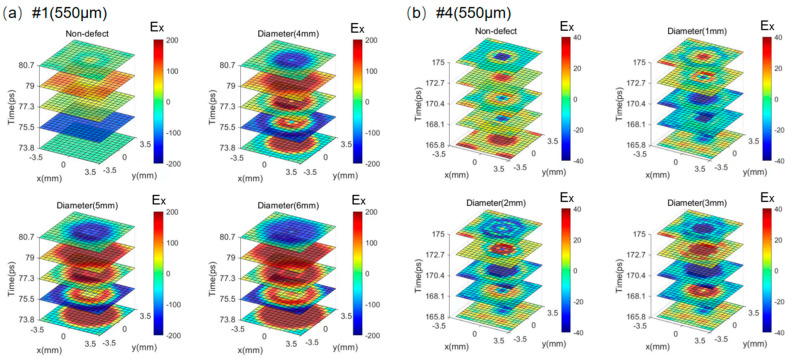
The simulated scanning images for (**a**) the top (#1–550 μm) and (**b**) the middle defects (#4–550 μm) in the sandwich structure with non-defect, 4 mm defect, 5 mm defect, and 6 mm defect.

At last, to reveal the defect shape more accurately and comprehensively, imaging technology is necessary. We simulate images of 15 × 15 pixels with a pixel size of 0.5 mm × 0.5 mm, which are enough to cover a whole honeycomb cell, for defect disks with different diameters. Figure 8 illustrates time-slice images for GFRP defects #1 (550 μm) and #4 (550 μm), which are representative of the applications. The defects in the bottom GFRP panel, as #7, can be transformed into the top ones by reversing the entire structure. Figure 8a ranges from 73.8 ps to 80.7 ps, where the feature reflections of the top layer defect are shown as a function of the *x*- and *y*-axes by the THz reflection amplitude signals. Compared to the time-slice images of the non-defect, the images of defect structures are obviously different, and the patterns of each layer, except for the 79 ps, are consistent very well with the corresponding defect diameters of 4 mm, 5 mm, and 6 mm. On the other hand, the time-slice images of the non-defect and middle defects with diameters of 1 mm, 2 mm, and 3 mm are illustrated in Figure 8b. The pixels in the images are generated by the THz reflection amplitude signals of time-slices ranging from 160 ps to 166.9 ps. Similarly, compared to the non-defective structures, the image contents of the defective disk structures are well consistent with the diameters except for 160 ps.

**Figure 9:**
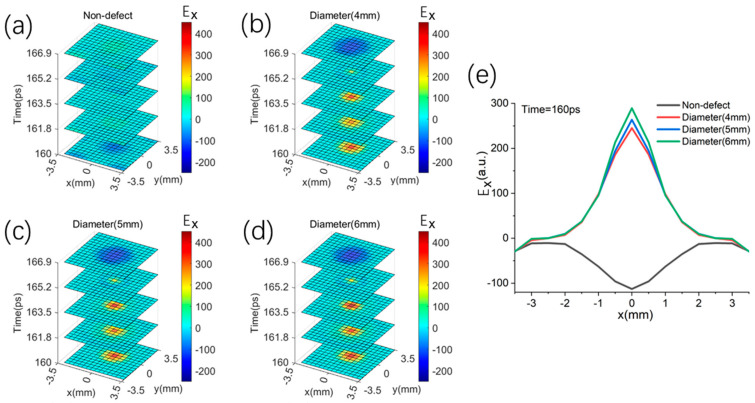
The simulated scanning images for the middle defect sandwich structure (#4–550 μm) by the THz reflection pulses. (**a**) non-defect; (**b**) diameter (4 mm); (**c**) diameter (5 mm); (**d**) diameter (6 mm); (**e**) the comparison of different image slices along the x-axis.

The larger defects of 4 mm, 5 mm, and 6 mm in the middle layer are a different situation. Due to the limit of the honeycomb core layer, the time-slice images of middle defects with diameters of 4 mm, 5 mm, and 6 mm are relatively very close, as shown in Figure 9b–d. To reveal the subtle image discrepancies, the image slices along the x-axis are demonstrated in Figure 9e. The slice profiles of three defects are different between −1 mm and 1 mm along the x-axis, but very consistent at other distances because of the waveguide effect of the aramid paper honeycomb cell.

In the summary, the results confirm that the scanning images at a specific time slice can be effective tools to detect the defect’s position and shape.

## 4. Conclusions

This study has numerically demonstrated the feasibility of THz-TDS detecting defects in sandwich GFRP panels and resins as supporting components for the aramid honeycomb structure with complex two aramid paper layers. The THz pulse reflection signals of defects in the GFRP panel and resin are carefully simulated using the FDTD method with a THz incident beam of a real pulse waveform. The results showed that the pulse of the THz-TDS system can clearly detect different positions and sizes of void defects, such as debonding and layering, with 1 mm−6 mm in diameter and 100 μm in thickness. By subtracting the original non-defect signal in the cell center, amplified differential signals of defects can more effectively reveal their positions and shapes. Furthermore, time-slice images ranging from 160 ps to 166.9 ps, where the feature reflections of the middle layer defect are shown as a function of the *x*- and *y*-axes by the electrical field amplitude of the THz reflection signals. The initial simulation research indicates that THz-TDS can be used to measure the structural positions and shapes of defects in panels and resins of aramid honeycomb sandwich with imaging technology in a C-scanning way, which is of great significance in practical applications.

## Figures and Tables

**Table 1:** The peak times and amplitudes of the defects with diameters of 4 mm, 5 mm, and 6 mm.

Defect Position	Peak Time (4 mm)	Peak Time (5 mm)	Peak Time (6 mm)	Peak Amplitude (4 mm)	Peak Amplitude (5 mm)	Peak Amplitude (6 mm)
#1 (150 μm)	66.1 ps	66.0 ps	66.1 ps	0 dB	0 dB	0 dB
#1 (550 μm)	71.8 ps	71.8 ps	71.8 ps	−2.52 dB	−2.41 dB	−2.29 dB
#1 (950 μm)	77.5 ps	77.5 ps	79.2 ps	−5.91 dB	−5.32 dB	−5.23 dB
#2 (Resin)	79.0 ps	78.9 ps	78.8 ps	−8.79 dB	−8.76 dB	−8.65 dB
#3 (Resin)	152.1 ps	152.3 ps	149.0 ps	−26.48 dB	−26.42 dB	−26.39 dB
#4 (150 μm)	153.2 ps	153.1 ps	151.5 ps	−23.76 dB	−23.73 dB	−23.30 dB
#4 (550 μm)	160.3 ps	160.5 ps	157.0 ps	−25.81 dB	−25.16 dB	−25.03 dB
#4 (950 μm)	165.9 ps	166.2 ps	166.6 ps	−26.94 dB	−26.76 dB	−26.35 dB
#5 (Resin)	167.6 ps	167.8 ps	166.2 ps	−26.78 dB	−28.65 dB	−28.44 dB
#6 (Resin)	243.9 ps	245.5 ps	237.7 ps	−39.73 dB	−39.42 dB	−39.30 dB
#7 (150 μm)	244.9 ps	252.0 ps	248.9 ps	−36.65 dB	−36.33 dB	−36.26 dB
#7 (550 μm)	252.3 ps	252.2 ps	252.3 ps	−36.81 dB	−36.79 dB	−36.75 dB
#7 (950 μm)	247.8 ps	250.0 ps	250.1 ps	−35.78 dB	−35.88 dB	−35.02 dB

## Data Availability

Data underlying the results presented in this paper are not publicly available but can be obtained from the authors upon reasonable request.
